# Reply to Niebuhr et al.: Infrastructure impacts must always be assessed locally

**DOI:** 10.1073/pnas.2214469119

**Published:** 2022-11-21

**Authors:** Sebastian Dunnett, Robert A. Holland, Gail Taylor, Felix Eigenbrod

**Affiliations:** ^a^Geography and Environmental Science, Faculty of Environmental and Life Sciences, University of Southampton, Southampton SO17 1BJ, United Kingdom; ^b^Biological Sciences, Faculty of Environmental and Life Sciences, University of Southampton, Southampton SO17 1BJ, United Kingdom; ^c^Plant Sciences, University of California, Davis, CA 95616

We thank Niebuhr et al. (1) for their important contribution to the discussion: We absolutely agree that measures of area overlap insufficiently handle the complexity of socioecological systems. As with Pérez-García et al. (2), Niebuhr et al. provide illuminating examples of exactly the “appropriate policy and regulatory” controls we call for in our paper to ensure true “minimal overlap” (3).

Our analysis narrowly focused on direct land take as it is an oft-used stick with which to beat proponents of energy system decarbonization; see, for example, recent comments by politicians in the United Kingdom (3) about field-scale solar taking land from agricultural production when the former comprises 0.1% of UK land area (4) and the latter, by some estimates, 70%. As such, we felt that it was important to emphasize that at global and regional scales, there appears to be ample scope for expanding both land uses if planned carefully.

We thank Niebuhr et al. for highlighting the importance of zones of influence and cumulative impacts. The median landscape area of global wind farms is 6.73 km^2^ (5), including an 800-m buffer around the outermost turbines, which makes the conservative distances used by Niebuhr et al. entirely appropriate. These increased impacts over a wider area make the colocation of renewable infrastructure with other damaging land uses, such as agriculture for wind and extant infrastructure for solar, even more important. Our analysis suggests, fortunately, that renewable energy siting—especially solar—is driven by many anthropogenic drivers over pure resource efficiency (6) (SI Appendix), indicating that this colocation is already common.

Niebuhr et al. highlight the significance of infrastructure impacts on indigenous communities. Although we looked at the sustainability nexus through energy and nature lenses, it is of course crucial that the achievement of these goals includes the explicit participation of those most affected to ensure social, as well as environmental, justice. Indigenous communities’ contribution to conservation is finally being recognized (7, 8). Renewable energy, done well with effective local participation, can similarly bolster local communities through energy decentralization and independence.

Infrastructure impacts must always be assessed at the local scale, considering potential cumulative regional impacts. We feel that our analysis presents evidence that cumulatively, there is enough land for locally appropriate deployment of wind and solar alongside biodiversity conservation. Our analysis is therefore intended to complement local assessment, facilitating avoidance of impacts where they are deemed by local assessment to be significant (cf. our response to Pérez-García et al. (9)). Yes, minimal overlap does not mean minimal impact, but it does significantly increase its likelihood.
